# Wheat responses to sodium vary with potassium use efficiency of cultivars

**DOI:** 10.3389/fpls.2014.00631

**Published:** 2014-11-11

**Authors:** Karthika Krishnasamy, Richard Bell, Qifu Ma

**Affiliations:** School of Veterinary and Life Sciences, Murdoch UniversityPerth, WA, Australia

**Keywords:** K–Na interaction, K uptake, K-use efficiency, Na uptake, wheat cultivars

## Abstract

The role of varied sodium (Na) supply in K nutrition of wheat (*Triticum aestivum* L.) is not well understood especially among cultivars differing in K efficiency. We examined the response of K-efficient and K-inefficient Australian wheat cultivars to Na supply (low to high Na) under K-deficient and K-adequate conditions. In a pot experiment, wheat *cvv* Wyalkatchem, Cranbrook (K-efficient), and *cvv* Gutha, Gamenya (K-inefficient) were grown for 8 weeks in a sandy soil containing 40 or 100 mg K/kg in combination with nil, 25, 50, 100, or 200 mg Na/kg. High soil Na levels (100, 200 mg Na/kg) greatly reduced plant growth in all four cultivars especially at low soil K (40 mg K/kg). By contrast, low to moderate soil Na levels (25, 50 mg Na/kg) stimulated root dry weight at low K supply, particularly in K-efficient cultivars compared with K-inefficient cultivars. At low K supply, low to moderate Na failed to increase shoot Na to a concentration where substitution of K would be feasible. However, low to moderate Na supply increased shoot K concentration and content in all four wheat cultivars, and it increased leaf photosynthesis and stomatal conductance to measured values similar to those under adequate K and nil Na conditions. The results showed that low to moderate Na stimulated K uptake by wheat particularly in K-efficient cultivars and through increased shoot K enhanced the photosynthesis. We conclude that increased photosynthesis supplied more assimilates that led to increased root growth and that greater root growth response of K-efficient cultivars is related to their greater K-utilization efficiency. However, the process by which low to moderate Na increased shoot K content warrants further investigation.

## INTRODUCTION

Key physiological roles for K are in stomatal regulation and in photosynthesis (Römheld and Kirkby, 2010). Sodium can substitute for non-specific biophysical functions of K^+^, especially where plants have the ability to take up, translocate, and compartmentalize Na in their vacuoles where it can replace functions of K in maintaining cell turgor (Subbarao et al., 2003; Gattward et al., 2012). In K-deficient soils, Na can play the role of K in maintaining ionic balance (Subbarao et al., 2003), regulating osmotic pressure (Marschner, 1995), provide partial K-substitution in protein synthesis (Flowers and Dalmond, 1992), contribute to vacuolar functions (Mäser et al., 2002), and improve water balance via regulation of stomatal conductance (Marschner, 1995; Gattward et al., 2012). Under K-deficiency, the addition of Na replaced K in stomatal functions of sugar beet and reduced the effects of K-deficiency on photosynthetic or respiratory CO_2_ exchange (Terry and Ulrich, 1973), and in net photosynthetic rate of *Theobroma cacao* (Gattward et al., 2012). Also under water deficit, stomata of sugar beet leaves supplied with Na closed more rapidly but exhibited delay in opening compared to supply of K only (Mäser et al., 2002). Sodium is also reported to alleviate effects of K-deficiency on plant growth in sugar beet, lettuce, cotton, rye grass, spinach, marigold, tomato (Mundy, 1983; Marschner, 1995; Tahal et al., 2000; Idowu and Aduayi, 2007; Pi et al., 2014), and barley (Ma et al., 2011). However, in wheat which maintains a high selectivity of K^+^ uptake relative to Na^+^ uptake, there are few reports of Na substitution for K. Box and Schachtman (2000) investigated whether Na supply can benefit wheat growth under low K by stimulating K^+^ uptake through the Na^+^ energized HKT1 symporter and found that low concentrations of Na^+^ did not increase K^+^ uptake to a large extent and while Na^+^ stimulated wheat growth at low external K it was only when light levels were low. By contrast, Marschner (1995) classified wheat as having moderate response to low Na. Hence further investigation is needed to clarify the response of wheat to low Na levels especially under low K.

Varieties of the same species can vary in K accumulation and utilization, e.g., sweet potato (George et al., 2002), cotton (Ali et al., 2006), and wheat (Damon and Rengel, 2007). Genotypic differences in cotton (Ali et al., 2006) and sugar beet (Marschner et al., 1981) are also reported in terms of Na substitution of K functions. Ali et al. (2006) studied 30 cotton genotypes in hydroponics and found that the genotypes differed significantly in growth responses, K uptake, K-use efficiency, and K substitution by Na. However, there is a lack of information about how cultivar variation in K-use efficiency alters effects of low to moderate soil Na on plant K nutrition. Understanding K and Na interactions among wheat cultivars that vary in K-use efficiency would improve management of K fertilizer in sodic and K-deficient soils.

We hypothesized that if high K-efficiency in wheat was related to higher K uptake, K-efficient cultivars would exhibit not only reduced salinity effects but also a reduced need for Na-substitution of K in plants. Alternatively, if greater Na substitution of K was the main mechanisms for greater K-use efficiency such cultivars could be more susceptible to salinity and demonstrate greater response to low to moderate Na levels in low soil. We examined the effect of Na levels on K uptake, the K^+^/Na^+^ ratios, leaf gas exchange, and plant growth of wheat cultivars differing in K-use efficiency. Supply of NaCl ranged from low to moderate levels, designed for substitution of K by Na at low K supply, ranging to toxic levels for wheat at high Na.

## MATERIALS AND METHODS

Wheat (*Triticum aestivum* L.) *cvv* Wyalkatchem, Cranbrook, Gutha, and Gamenya were grown in a naturally lit glasshouse at Murdoch University, Perth (32°04′S, 115°50′E) from late winter to mid spring. The average minimum and maximum temperatures during the experiment were 8.4 and 26°C, respectively. Cultivars Wyalkatchem and Cranbrook are K-efficient, whereas Gutha and Gamenya are K-inefficient in terms of K uptake and use (Damon and Rengel, 2007). The sandy soil was collected from an unfertilized field, 150 km northeast to Perth, and had the following properties: pH 4.9 (0.01 M CaCl_2_), EC_1:5_ 0.03 dS/m, 7 mg NH_4_-N/kg and 9 mg NO_3_-N/kg (Searle, 1984), <15 mg K/kg and 29 mg P/kg (Colwell, 1963) and organic C 0.17% (Walkley and Black, 1934).

Sieved soils (<2 mm) were well mixed with basal nutrients and individual treatments of K and Na, and filled into undrained plastic pots (diameter 190 mm, depth 190 mm) at 6 kg/pot. Basal nutrients were applied at the following rates (mg/kg): 103 (NH_4_)_2_HPO_4_, 237 Ca(NO_3_)_2_.4H_2_O, 80 MgSO_4_.7H_2_O, 18 FeSO_4_.7H_2_O, 14 MnSO_4_.H_2_O, 9 ZnSO_4_.7H_2_O, 8.3 CuSO_4_.5H_2_O, 0.33 H_3_BO_3_, 0.3 CoSO_4_.7H_2_O, 0.33 Na_2_MoO_4_.2H_2_O. Seeds were washed with 5% (w/w) hypochlorite solution for 1 min, thoroughly rinsed and soaked in de-ionised (DI) water for 2 h, and then placed in a refrigerator at 4°C overnight. The sprouted seeds were transferred to trays containing 0.05 mM CaCl_2_ solution, and covered for 2 days. Five germinated seeds per pot were transplanted, and 10 days later the seedlings were thinned to three plants per pot. During the experiment, the pot soils were watered daily to field capacity (15% w/w) with DI water. The plants were supplied with 0.5 mM urea solution fortnightly to maintain adequate N supply. The pots were re-arranged every week to reduce positional effects on plant growth.

### POTASSIUM AND SODIUM TREATMENTS

Two soil K levels were applied: 40 mg K/kg (low) and 100 mg K/kg (adequate) based on earlier trials (Ma et al., 2011). Muriate of potash (KCl) was used as it is the dominant K fertilizer (Moore, 2004). Each soil K level also included five Na levels: nil, 25, 50, 100, and 200 mg Na/kg supplied as NaCl. Equivalent Na concentrations in soil solution were 0, 7.25, 14.5, 29.1, and 58.2 mM, respectively, while ECe (electrical conductivity of saturated soil extract) values for Na treatments at 40 K were 0.85, 1.26, 2.66, 5.18, and 10.9 dS/m and at 100 K were 1.23, 1.82, 3.78, 7.28, and 13.7 dS/m. Therefore, the experiment comprised a factorial combination of four wheat cultivars, two K levels, and five Na levels. All the treatments were replicated three times. At potting, individual treatments with various K and Na levels were mixed thoroughly with basal nutrients using a rotary mixer.

### MEASUREMENTS

Plants were grown for 8 weeks and during that period the number of leaves and tillers was recorded weekly. Leaf net photosynthesis, transpiration and stomatal conductance were measured using the LCpro^+^ advanced photosynthesis system (ADC Bioscientific, UK) at 7 weeks after transplanting. The measurements were made in fully expanded young leaves at ambient relative humidity of 50%, leaf temperature of 25°C, reference CO_2_ of 380 μmol/mol, and photosynthetically active radiation of 1500 μmol/m^2^ s^1^.

At harvest, the shoot was cut at the soil surface, and the fresh weight was recorded immediately. Roots were collected after washing in tap water and rinsing in de-ionised (DI) water. The shoot and root samples were dried in a forced-draft oven at 60°C for 48 h and dry weight was recorded. About 0.2 g of each milled sample was weighed into centrifuge tubes and digested in 5 mL 70% (w/w) HNO_3_ at 75°C for 10 min, and then at 109°C for 15 min. After the samples were cooled, 1 mL of 30% (w/w) H_2_O_2_ was added and further digested at 109°C for 15 min. The digestion was made in a micro-wave oven (CEM Mars 5, CEM Corp., USA) based on method of Huang et al. (2004) for cation analysis. The digested samples were diluted with milli-Q water and concentrations of K, Na, Ca, and Mg were determined by inductively coupled plasma-atomic emission spectroscopy (VISTA Simultaneous ICP-AES, Varian). The K^+^/Na^+^ ratios in shoots and roots were calculated based on their content.

A supplementary study was conducted to determine whether the extractable cation levels, particularly K, were influenced by different soil Na levels. Two kilograms of soils were thoroughly mixed with basal nutrients and two K levels (40, 100 mg K/kg). Each soil K level was treated with two levels of Na: nil, 50 mg Na/kg. The pots were watered with DI water to field capacity, and allowed to equilibrate for a week, while mixing daily. The soil samples were then analyzed for bicarbonate-extractable (Colwell, 1963) K and exchangeable cations.

### STATISTICAL ANALYSIS

Statistical analyses were conducted using the SPSS statistical package (IBM SPSS statistics, vs. 18). Three-way analysis of variance was conducted to assess the effects of soil K and Na supply, genotype and their interactions. Tukey’s HSD was computed at *P* ≤ 0.05 to test for differences among treatment means.

## RESULTS

### PLANT GROWTH

#### Shoot growth

**FIGURE 1:**
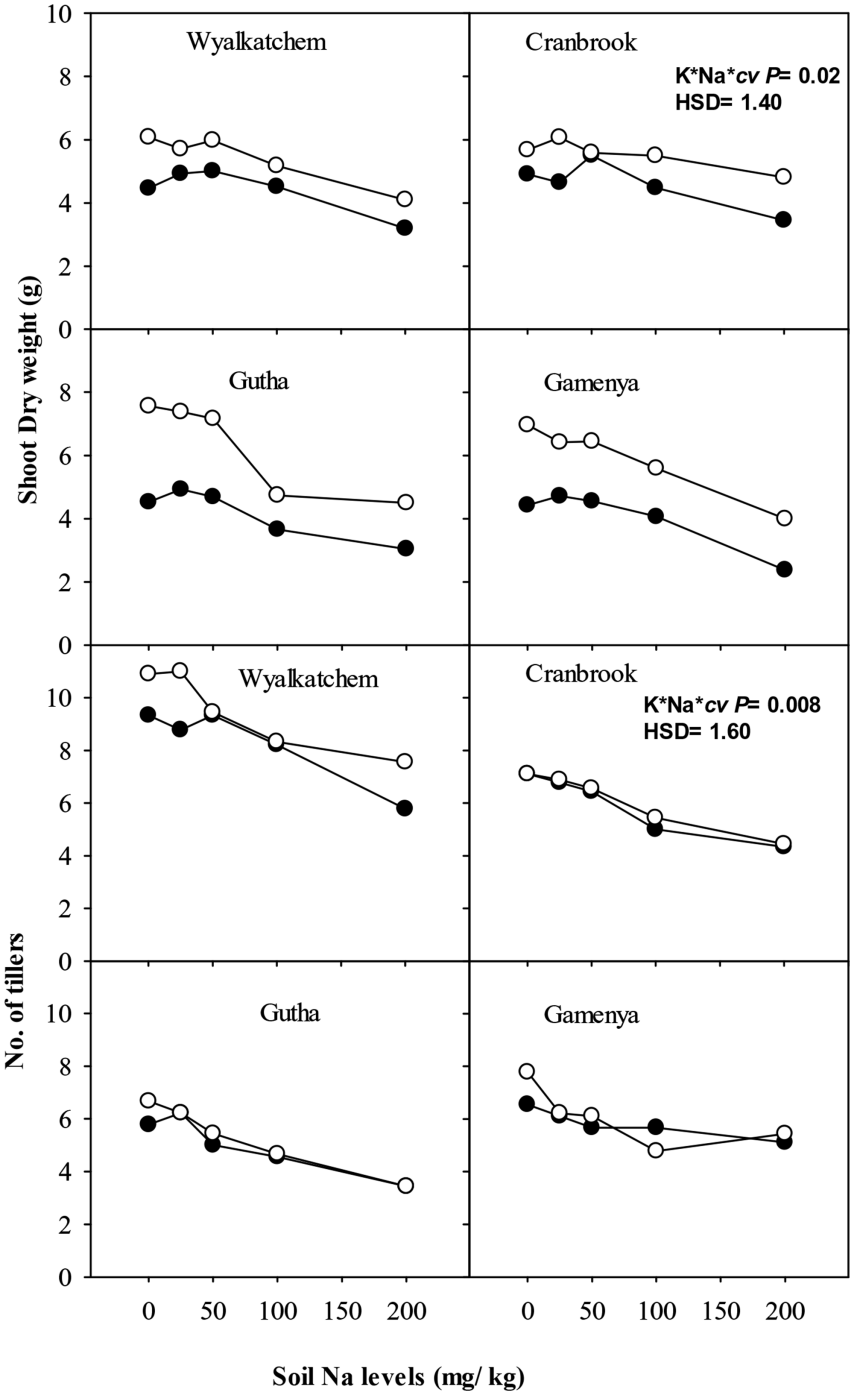
**Shoot dry weight (g/plant; upper sub-figures), and tillers/plant (lower sub-figures; *n* = 3) of four wheat cultivars, treated with 40 mg K/kg (closed circle) and 100 mg K/kg (open circle), and five soil Na levels (0, 25, 50, 100, and 200 mg Na/kg) for 8 weeks.** See **Table 1** for analysis of variance results.

Low K supply (40 mg K/kg) induced K-deficiency symptoms after 6 weeks and significantly reduced shoot dry weight at 8 weeks, but the reduction was greater in K-inefficient cultivars Gutha and Gamenya (32% lower) than K-efficient cultivars Wyalkatchem and Cranbrook (17–18% lower). When K supply was low, the addition of low to moderate Na (25, 50 mg Na/kg) alleviated K-deficiency symptoms in old leaves but had no significant effects on shoot dry weight (**Figure 1**). Similarly, at adequate K supply, addition of 25–50 mg Na/kg had no effect on shoot dry weight. High soil Na levels (100, 200 mg Na/kg) reduced shoot dry weight especially in K-inefficient cultivars (**Figure 1**). When compared with nil Na, high Na reduced shoot dry weight by 44% in Gamenya, 38% in Gutha, 31% in Wyalkatchem and 22% in Cranbrook. The interactions between K, Na and cultivars on shoot dry weight were significant (*P*≤ 0.05; **Table 1**).

**Table 1:** Statistical summary of plant growth and leaf gas exchange in four wheat cultivars (Wyalkatchem, Cranbrook, Gutha, and Gamenya) treated with two K levels (40, 100 mg K/kg) and five Na levels (0, 25, 50, 100, 200 mg Na/kg) for 8 weeks.

Parameters	Soil K	Soil Na	Cultivar	K × Na	K × *cv.*	Na × *cv.*	K × Na × *cv.*
Shoot dry weight	***	***	*	**	***	***	*
Tiller number	***	***	***	*	***	***	**
Root dry weight	***	***	***	*P*= 0.06	*	***	n.s
Root: shoot ratio	**	***	***	n.s	*	***	n.s
Photosynthesis	***	***	***	***	n.s	*	***
Stomatal conductance	***	***	**	*	n.s	n.s	*

*P≤ 0.05; **P≤ 0.01; ***P≤ 0.001; n.s., not significant.

Adequate soil K produced the same number or more tillers than low K at all soil Na levels, except in Gamenya that had fewer tillers at 100 mg Na/kg. Compared with Wyalkatchem, fewer tillers per plant were produced in *cvv* Gutha and Gamenya (**Figure 1**). Plants treated with low to moderate soil Na (25, 50 mg Na/kg) had similar tiller number as those of nil Na plants. However, high Na reduced tillers significantly in all four cultivars (*P* ≤ 0.05; **Table 1**).

#### Root growth

**FIGURE 2:**
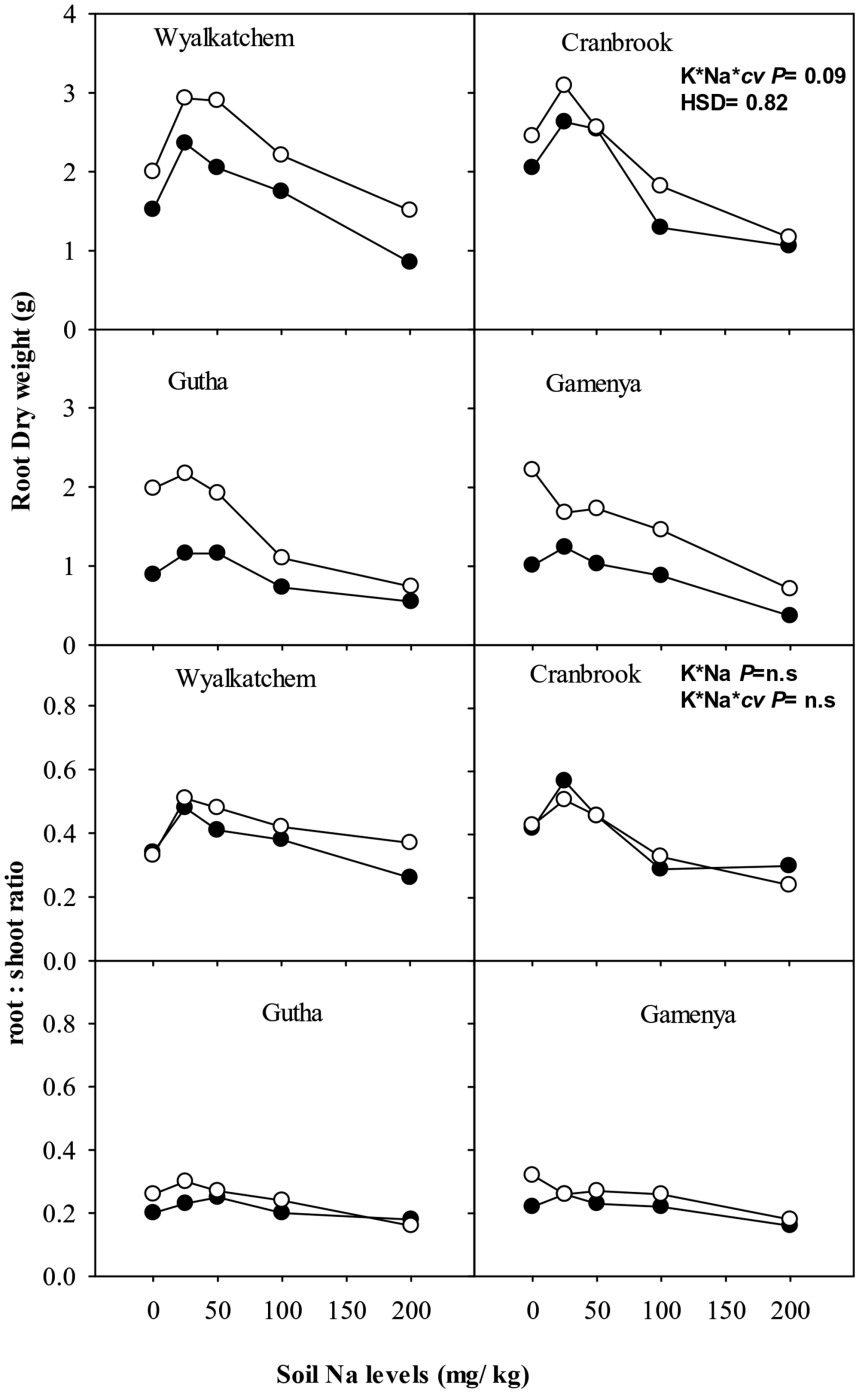
**Root dry weight (g/plant; upper sub-figures) and root: shoot ratio (lower sub-figures; *n* = 3) of four wheat cultivars, treated with 40 mg K/kg (closed circle) and 100 mg K/kg (open circle), and five soil Na levels (0, 25, 50, 100, and 200 mg Na/kg) for 8 weeks.** See **Table 1** for analysis of variance results.

Root dry weight of all four cultivars was greater at adequate K than at low K supply, regardless of soil Na levels (**Figure 2**). Low to moderate soil Na had positive effects on root dry weight in all four cultivars when soil K was low, and even at adequate K supply low soil Na was beneficial to root dry weight except in Gamenya (**Figure 2**). The Na-induced root stimulation was greater in K-efficient cultivars. High soil Na levels suppressed root dry weight in all four cultivars at both soil K levels, with greater reduction of root dry weight in K-inefficient cultivars (55% in Gutha and 66% in Gamenya) than in K-efficient cultivars (33% in Wyalkatchem, 50% in Cranbrook). In general, low K plants had lower root: shoot ratios compared with K-adequate plants, except Cranbrook at low Na (**Figure 2**), however, the interactions between K and Na for root: shoot ratio was not significant (**Table 1**).

### LEAF GAS EXCHANGE

**FIGURE 3:**
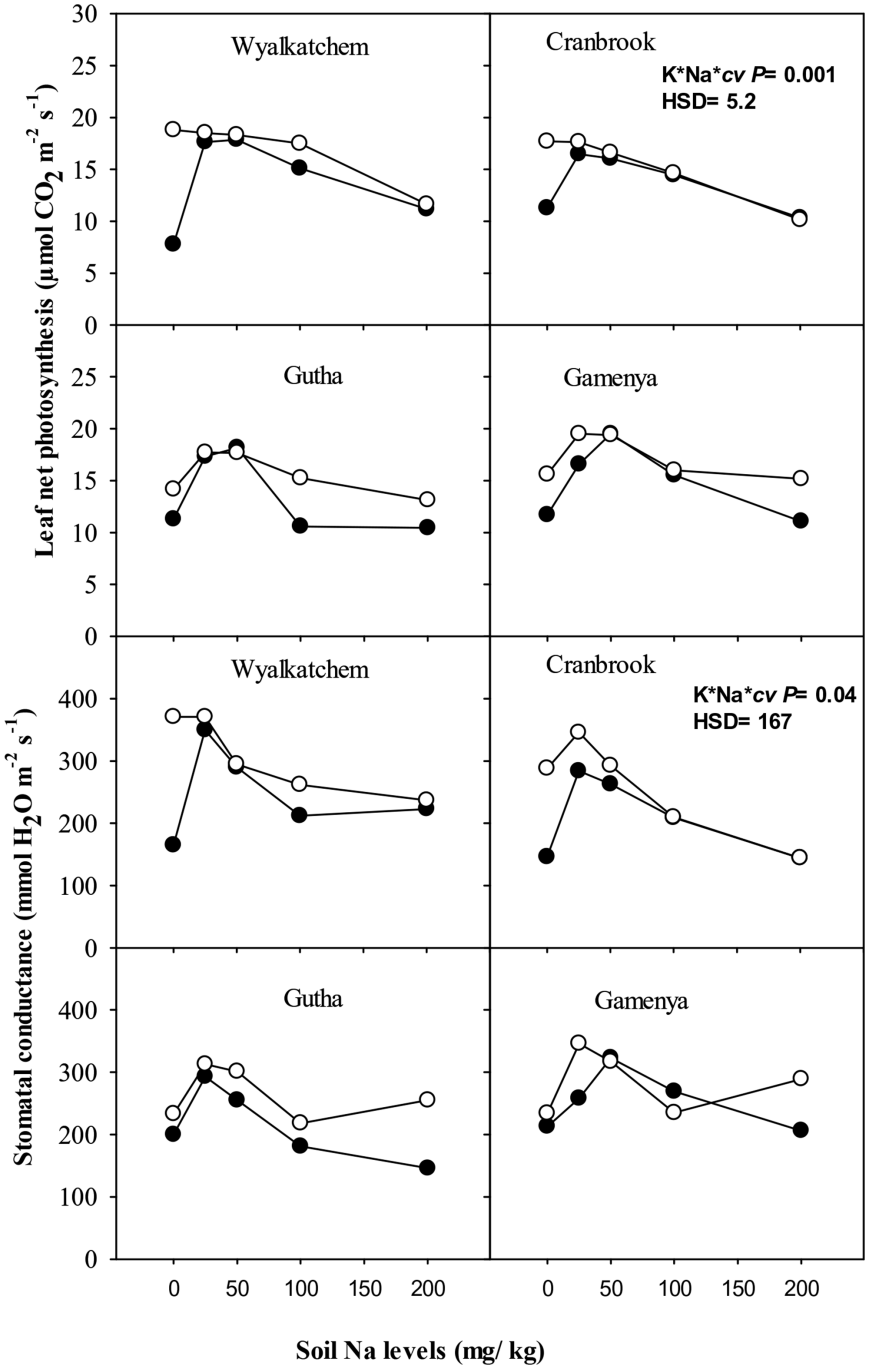
**Leaf photosynthesis (upper sub-figures) and stomatal conductance (upper sub-figures; *n* = 3) of four wheat cultivars, treated with 40 mg K/kg (closed circle) and 100 mg K/kg (open circle), and five soil Na levels (0, 25, 50, 100, and 200 mg Na/kg) for 8 weeks.** See **Table 1** for analysis of variance results.

At 4 weeks, low K depressed net photosynthesis of the youngest fully expanded leaves but there was no effect of low to moderate Na on gas exchange. At 8 weeks, consistent with the shoot dry weight responses, net photosynthesis of the youngest fully expanded leaves was higher in plants with adequate K than low K supply, except in Gutha at 50 mg Na/kg (**Figure 3**). The addition of 25 and 50 mg Na/kg increased leaf photosynthesis in all cultivars at low soil K and also in the K-inefficient cultivars at adequate K supply, whereas higher soil Na suppressed leaf photosynthetic rate relative to addition of 25 and 50 mg Na/kg. The increase in leaf net photosynthesis induced by 25–50 mg Na/kg at low K was almost equal to that at 100 mg K/kg and nil Na. There were significant interactions between soil K, Na and cultivars for leaf photosynthesis (*P*≤ 0.05; **Table 1**). Similarly, stomatal conductance of low K plants increased with the addition of 25 mg Na/kg in all cultivars (**Figure 3**). Higher soil Na reduced stomatal conductance in the K-efficient cultivars but was less so in the K-inefficient cultivars. At low K supply, the addition of low to moderate soil Na increased transpiration rate of K- efficient cultivars by 54%, whereas in K-inefficient cultivars the increase was only by 35% relative to nil Na (data not presented).

### K AND Na CONCENTRATIONS IN SHOOTS AND ROOTS

**FIGURE 4:**
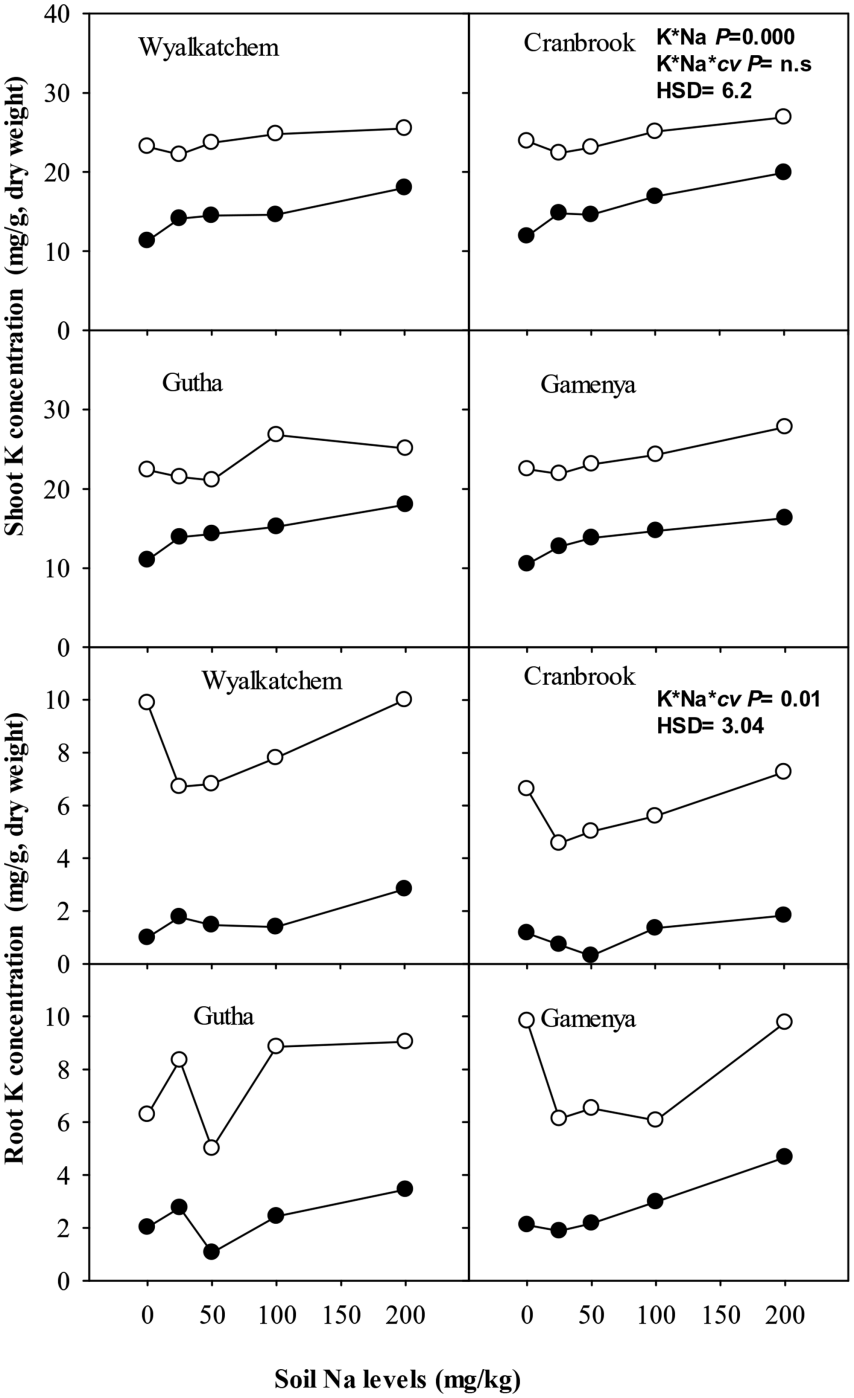
**Potassium (K) concentration (mg/g, dry weight) in shoot (upper sub-figures) and root (lower sub-figures; *n* = 3) of four wheat cultivars, treated with 40 mg K/kg (closed circle) and 100 mg K/kg (open circle), and five soil Na levels (0, 25, 50, 100, and 200 mg Na/kg) for 8 weeks.** See **Table 2** for analysis of variance results.

Potassium concentration in leaves and stems of all four cultivars was significantly higher with adequate K than low K supply when soil Na levels ranged from nil to moderate, whereas spikes had similar K concentrations irrespective of K and Na treatments (data not presented). At low K supply, plants with nil Na treatment had the lowest shoot K concentration in all cultivars (**Figure 4**). Low to moderate Na supply increased shoot K concentrations of the four cultivars on average by 25% relative nil Na at low K supply (**Table 2**). High soil Na also increased shoot K concentrations with both low and adequate soil K supply in all cultivars, but probably due to a concentration effect as a result of growth suppression.

**Table 2:** Statistical summary of K and Na concentrations and contents in four wheat cultivars (Wyalkatchem, Cranbrook, Gutha, and Gamenya) treated with two levels of soil K (40, 100 mg K/kg) and five levels of Na (0, 25, 50, 100, 200 mg Na/kg) for 8 weeks.

Parameters	Soil K	Soil Na	Cultivar	K × Na	K × *cv.*	Na × *cv.*	K × Na × *cv.*
K concentration shoot	***	***	*P = 0.09*	**	n.s	n.s	n.s
K concentration root	***	***	***	***	***	***	**
Shoot K content	***	***	***	***	***	*	**
Root K content	***	***	***	***	***	***	***
Na concentration shoot	**	***	*P = 0.06*	*P = 0.07*	*P = 0.09*	n.s	n.s
Na concentration root	**	***	***	*P = 0.07*	***	***	***
Shoot Na content	n.s	***	*P = 0.06*	n.s	*P = 0.06*	n.s	n.s
Root Na content	***	***	***	***	n.s	***	n.s
Shoot K^+^/Na^+^	***	***	**	***	*	n.s	n.s
Root K^+^/Na^+^	***	***	n.s	***	n.s	n.s	n.s

*P≤ 0.05; **P≤ 0.01; ***P≤ 0.001; n.s., not significant.

**FIGURE 5:**
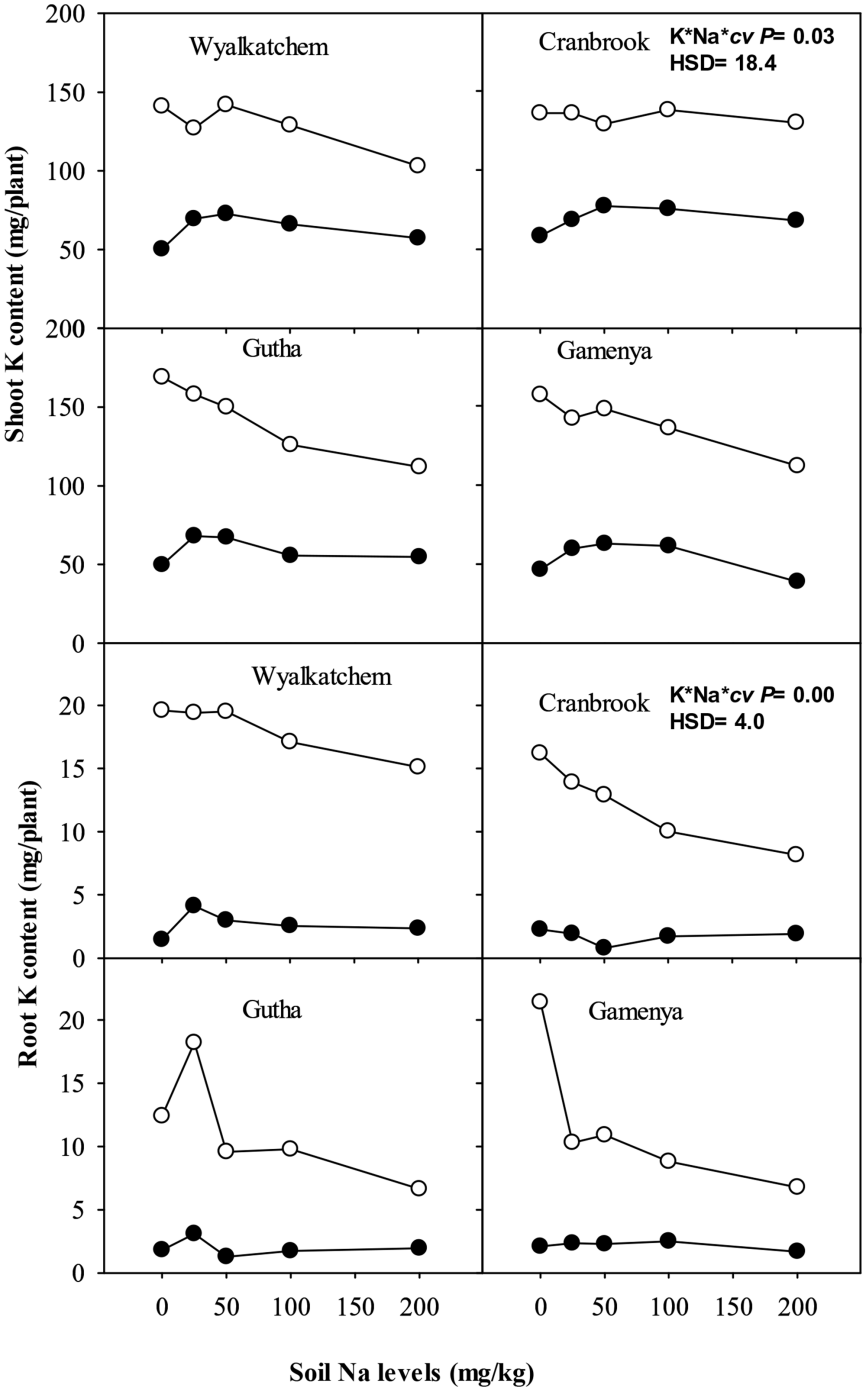
**Potassium (K) content (mg/plant) in shoot (upper sub-figures) and root (lower sub-figures; *n* = 3) of four wheat cultivars, treated with 40 mg K/kg (closed circle) and 100 mg K/kg (open circle), and five soil Na levels (0, 25, 50, 100, and 200 mg Na/kg) for 8 weeks.** See **Table 2** for analysis of variance results.

Although shoot K content was much greater in the adequate K soil than in the low K soil, it showed little difference or declined across soil Na levels at the high level of soil K (**Figure 5**). At low soil K supply (40 mg K/kg), shoot K contents increased significantly with low to moderate soil Na addition in K-efficient cultivars but not in K-inefficient cultivars (**Figure 5**). There were significant interactions of soil K, Na supply and cultivars on shoot K contents (*P*≤ 0.05; **Table 2**).

Wheat roots accumulated considerably less K^+^ than shoots. Root K concentration and content of all cultivars was significantly higher at adequate K supply than with low K supply (**Figures 4** and **5**). At low K supply, soil Na addition had no significant effect on root K content (**Figure 5**). At adequate K supply, there was decrease in root K content with addition of soil Na, except in Gutha at 25 mg Na/kg which showed a significant increase, and the decrease due to Na was more obvious in K-inefficient *cv.* Gamenya. The three way interaction between soil K and Na levels and cultivars was significant (*P*≤ 0.05, **Table 2**) for root K concentrations and contents.

In all cultivars, shoot Na concentrations were closely associated with soil Na levels (**Table 3**). Old leaves and stem concentrated at least four times more Na than young leaves. Sodium concentration in spikes was least influenced by soil Na irrespective of soil K and cultivars, and there were only negligible concentrations of Na measured in spikes (data not presented). Soil K levels did not influence shoot Na concentration and content. There were no significant interactions observed between K and Na supply for shoot Na concentrations and contents, nor among the cultivars (**Table 2**).

**Table 3:** Shoot and root Na concentrations (mg/g, dry weight) and contents (mg/plant) of four wheat cultivars treated with two K levels (40, 100 mg K/kg) and five Na levels (0, 25, 50, 100, 200 mg Na/kg) for 8 weeks (*n* = 3).

Measured parameters	*cvv*	Wyalkatchem		Cranbrook		Gutha		Gamenya	
	Na	K40	K100	K40	K100	K40	K100	K40	K100
Shoot Na concentration HSD_0.05_ = 1.5	Nil Na	0.09	0.15	0.11	0.08	0.12	0.13	0.11	0.12
	Na25	0.18	0.23	0.33	0.18	0.26	0.33	0.29	0.33
	Na50	0.61	0.49	0.82	0.42	0.82	0.54	1.07	0.49
	Na100	1.16	1.51	2.11	1.27	1.48	2.36	2.55	1.61
	Na200	2.76	2.72	3.71	2.81	4.00	2.89	3.34	2.71

Root Na concentration HSD_0.05_ = 3.57	Nil Na	2.74	1.56	1.76	1.32	3.07	1.32	2.92	1.32
	Na25	4.23	4.01	4.9	4.94	7.73	6.23	5.22	4.62
	Na50	7.72	8.47	7.14	8.79	9.57	7.48	9.21	8.93
	Na100	10.1	10.03	12.1	12.3	10.6	12.8	10.9	8.65
	Na200	16.5	12.9	15.4	18.5	16.5	14.6	18.4	15.6

Shoot Na content HSD_0.05_ = 6.2	Nil Na	0.42	0.91	0.52	0.49	0.52	0.99	0.47	0.83
	Na25	0.90	1.30	1.53	1.11	1.28	2.41	1.38	2.10
	Na50	3.05	2.92	4.51	2.33	3.82	3.93	4.91	3.21
	Na100	5.31	7.94	9.48	6.94	5.43	10.9	10.5	8.93
	Na200	8.77	10.9	12.6	13.5	12.1	13.1	8.04	11.0

Root Na content HSD_0.05_ = 6.3	Nil Na	4.13	3.18	3.44	3.22	2.68	2.62	2.89	2.95
	Na25	9.86	11.6	12.8	14.9	8.87	13.5	6.34	7.62
	Na50	15.9	24.1	17.9	22.4	11.0	14.5	9.50	15.2
	Na100	16.9	22.0	15.7	22.3	7.60	14.1	9.50	12.6
	Na200	14.1	19.4	16.0	21.6	9.19	10.6	6.79	10.9

*See **Table 2** for statistical summary of main effects and interactions of the treatments*.

In contrast to K, Na concentration and content in roots were much higher than in shoots (**Table 3**). Root Na concentrations rose with increase in soil Na levels. Similar to shoot, soil K levels had no influence on root Na concentration. However, root Na content was higher in plants grown at adequate K except at nil Na. The interactions between K and Na and cultivars were significant for root Na concentrations but not for Na contents (**Table 2**).

Shoot K^+^/Na^+^ ratios noticeably decreased with increase in soil Na levels in all four cultivars regardless of soil K levels (**Table 4**). At low soil K, Gamenya and Wyalkatchem had the lowest and highest shoot K^+^/Na^+^ ratios, respectively. However, at adequate K, Cranbrook had highest shoot K^+^/Na^+^ ratio at most of the soil Na levels. Roots had considerably lower K^+^/Na^+^ ratios than shoots. They decreased further with increasing soil Na levels but there was no particular trend observed among the cultivars. The interaction between K and Na supply on shoot and root K^+^/Na^+^ ratio was significant but not among the cultivars (**Table 2**).

**Table 4:** Shoot and root K^+^/Na^+^ ratios of four wheat cultivars treated with two K levels (40, 100 mg K/kg) and five Na levels (0, 25, 50, 100, 200 mg Na/kg) for 8 weeks (*n* = 3).

Measured parameter	*cvv*	Wyalkatchem		Cranbrook		Gutha		Gamenya	
	Treatment	K40	K100	K40	K100	K40	K100	K40	K100
Shoot K^+^/Na^+^ HSD_0.05_ = 81	Nil Na	123	196	115	279	90.5	177	104	196
	Na25	86.7	100	46.1	137	54.5	68.3	44.7	68.4
	Na50	24.5	48.9	17.1	56.5	17.7	39.3	12.9	47.2
	Na100	12.9	17.9	8.05	21.9	10.3	13.4	5.73	17.8
	Na200	6.63	10.04	5.93	9.71	4.55	8.78	4.98	10.4

Root K^+^/Na^+^ HSD_0.05_ = 2.5	Nil Na	0.36	7.03	0.67	5.49	0.72	5.56	0.72	7.53
	Na25	0.45	1.67	0.14	0.94	0.35	1.34	0.38	1.38
	Na50	0.19	0.81	0.04	0.57	0.11	0.69	0.24	0.72
	Na100	0.15	0.77	0.11	0.45	0.23	0.69	0.27	0.70
	Na200	0.17	0.78	0.12	0.39	0.21	0.62	0.25	0.62

*See **Table 2** for statistical summary of main effects and interactions of the treatments*.

### SOIL EXCHANGEABLE CATIONS AFTER K AND Na ADDITION

The soil incubation experiment did not show any significant effects of Na addition on exchangeable soil K levels or exchangeable Ca, Mg, and Al levels. Colwell-extractable K and exchangeable K measured were slightly higher when soil had Na applied, compared with nil soil Na, but the increase was not statistically significant (**Table 5**).

**Table 5:** Concentrations of exchangeable cations in non-planted soils (*n* = 3) with or without 50 mg Na/kg at two K levels (40, 100 mg K/kg) after 1 week of incubation.

	Soil treatment			
Measured parameters	K 40	K 40	K 100	K 100
	Nil Na	50 Na	Nil Na	50 Na
Colwell K (mg/kg)	48.3^b^	51.0^b^	96.0^a^	102.7^a^
Exchange K (cmol/kg)	0.13^a^	0.14^a^	0.24^b^	0.27^b^
Exchange Na (cmol/kg)	0.02^b^	0.19^a^	0.02^b^	0.18^a^
Exchange Ca (cmol/kg)	0.68^a^	0.71^a^	0.44^b^	0.61^ab^
Exchange Mg (cmol/kg)	0.09^a^	0.10^a^	0.10^a^	0.10^a^
Exchange Al (cmol/kg)	0.02^a^	0.03^a^	0.03^a^	0.03^a^

*Means with different letters differ at *P* ≤ 0.05.*

## DISCUSSION

Wheat response to soil NaCl supply in the present study varied with soil Na and K levels: (1) root growth was stimulated by low to moderate soil Na levels with low soil K; (2) shoot and root dry weight were suppressed with high Na regardless of soil K levels. However, the Na effect varied with K-use efficiency of wheat cultivars with K-efficient cultivars being more responsive to root dry weight stimulation by low to moderate Na under K deficiency along with greater increases in shoot K uptake and stomatal conductance than K-inefficient cultivars. Genotypic differences in K-use efficiency also influenced Na uptake and salt tolerance: K-efficient cultivars were more tolerant of high salt levels than K-inefficient cultivars.

The growth stimulation at low to moderate Na (25, 50 mg/kg) supply under K deficiency was clearly evident in wheat roots but in shoots only through the alleviation of old leaf K deficiency symptoms. The shoot dry weight and fresh weights were not significantly affected by low to moderate Na in K-deficient plants. However, Na at 100–200 mg/kg negatively affected both root and shoot dry weight of wheat with both low and adequate soil K due to salt toxicity. The present results in wheat were in contrast to salt tolerant barley where the addition of 100 mg Na/kg to K-deficient soil stimulated significant shoot growth increase but not in root growth (Ma et al., 2011). Moreover, Ma et al. (2011) found, similar to the present study, no significant benefit after 20 days of 100 mg Na/kg supply on K-deficient (30 mg K/kg) wheat growth. Indeed, 100 mg Na/kg (equivalent to about 30 mM Na in soil solution) for 8 weeks had negative effects on wheat growth in the current study. Clearly there were contrasting effects of low to moderate Na on wheat and barley. Barley responded positively to moderate Na (100 mg Na/kg) supplied to K-deficient plants, but the response was restricted to the shoots and not the roots. Wheat on the other hand only responded to Na at lower levels (25–50 mg Na/kg but not 100 mg Na/kg) with the strong response in roots but not in shoots.

Differences in accumulation of K^+^ and Na^+^ between barley and wheat may explain their contrasting responses to low to moderate Na supply. Wheat roots accumulated significantly higher Na than in shoots. Indeed the low to moderate level of Na that stimulated root growth, increased root Na from 3 mg Na/kg (at nil Na) to 9.5 and 14 mg Na/kg at 25 and 50 mg Na/kg respectively, while shoot Na only increased from 0.48 mg Na/kg to 1.3 and 4 mg Na/kg, at 25 and 50 mg Na/kg respectively. In the study of Ma et al. (2011), shoot K^+^/Na^+^ in wheat *cv.* Wyalkatchem was 10–15 times higher than the ratio in shoots of two barley cultivars grown under the same conditions. By contrast, there were no differences among barley cultivars and wheat in K^+^/Na^+^ ratio in roots. Hence, the high accumulation of Na in barley shoots produced a K^+^/Na^+^ ratio of 0.5 in barley *cv*. CM72 but 10 in wheat. Under these conditions, the substitution of K by Na is feasible in barley since there is sufficient Na^+^ to provide equivalent osmotic effects to those of K^+^. In barley the accumulation of Na^+^ by low-K plants was limited to shoots as was the shoot growth response. By contrast, the low shoot Na^+^ concentration in wheat shoots relative to K^+^, provides too little Na for the replacement of K functions in the shoot to be feasible. For example in the low K plants, shoot K concentration was 13.9 K mg g^-1^ dry weight (equivalent to 89.5 mM K in tissue water) at 25 mg Na/kg of soil, whereas, depending on cultivars there was only 0.18–0.33 mg Na g^-1^ dry weight (mean tissue water Na concentration of 2.8 mM). Hence, there may be other processes in wheat that led to growth stimulation at 25–50 mg Na/kg.

In both the present study, and that of Ma et al. (2011), low to moderate Na supply to low-K wheat plants increased shoot K concentrations and this should have stimulated growth. The average shoot K concentrations in low and adequate soil K treatments were 14 and 24 g/kg, which are in the deficient and sufficient ranges, respectively, for wheat growth at the boot to heading stage (Reuter et al., 1997). The increases in photosynthesis rate and stomatal conductance also evident with low to moderate Na supply to K-deficient are both expected responses in the shoot to increased K concentration (Zhao et al., 2001). The increase in root growth is another expected plant response to increased shoot K concentration because increased photosynthesis results in greater assimilate supply to roots and increases root: shoot ratio of cereals (e.g., Degl’Innocenti et al., 2009; Ma et al., 2011, 2013). Hence a possible explanation for the Na stimulation of growth in wheat is that Na increases K supply to the shoot which in turn stimulates photosynthesis and the greater supply of assimilate allows for increased root growth. With only a single harvest it is not possible to definitively piece together this chain of events. However, clearly the evidence in support of the first response, the increase in K uptake leading to greater shoot K is pivotal.

Increased shoot K content could arise from several mechanisms. Firstly, increase of root Na concentration at low to moderate Na may release vacuolar K^+^ that is made available for cytoplasmic functions in the root cells or for translocation to the shoot (Walker et al., 2000). The increase in root Na concentration at 25–50 mg Na/kg of soil was substantial, while root K contents remained unchanged. The effects of 25–50 mg Na/kg of soil on root K concentrations varied among cultivars. By contrast, shoot K content increased by about 40% with the supply of 25–50 mg Na/kg of soil. Hence the low to moderate Na supply appeared to favor K partitioning to the shoot of wheat.

A second possible mechanism for increased K uptake is Na activation of K^+^ symporters in roots. Under K deficiency, there is increased expression of the high-affinity K^+^ transporter (HKT; Anschütz et al., 2014). The HKT mediates high-affinity K^+^ uptake and high or low-affinity Na^+^ uptake depending on external Na^+^ and K^+^ concentrations (Benito et al., 2014). At low external Na^+^ and K^+^ concentrations, some transporters function as Na^+^–K^+^ symporters, as demonstrated by Na^+^-stimulated K^+^ uptake and K^+^- stimulated Na^+^ uptake, however, at high external Na^+^ concentrations, some of these transporters become Na^+^ uniporters, no longer transporting K^+^ (Rubio et al., 1995; Benito et al., 2014). Transporters of the HKT-type discriminate less between K^+^ and Na^+^ or even select for Na^+^ over K^+^ (Benito et al., 2014). However, Box and Schachtman (2000) reported the Na activation of K symporters increased K uptake only under low light conditions in wheat and concluded that it was functionally a minor process for K uptake by wheat. Nevertheless, the present study found K-use efficiency of wheat cultivars altered the response to low Na, indicating there may be effects of Na on transporters not identified by Box and Schachtman (2000).

An alternative mechanism for increased K^+^ uptake could be by a low-affinity K^+^ uptake system (such as AKT). At high Na levels (80 mM NaCl or above), Na^+^ crosses the plasma membrane causing a significant membrane depolarization and increases K^+^ leakage through depolarization-activated outward-rectifying channels (Shabala and Cuin, 2008). In sharp contrast to 80 mM NaCl treatment, K^+^ eﬄux in 20 mM NaCl treatment was found to be very short-lived and K^+^ uptake became dominant from the elusive ‘osmosensing mechanism’ (Chen et al., 2005). At moderate salinity (20 mM NaCl in barley), Na^+^ hyperpolarized the plasma membrane and increased K^+^ uptake via inward-rectifying hyperpolarized-activated K^+^ channels (Chen et al., 2005; Shabala and Cuin, 2008). Therefore, Na-induced K^+^ flux was clearly dose dependent, and could possibly explain increased K^+^ uptake at moderate Na levels.

A part but not the entire increase of K content could be attributed to the increase in extractable soil K by soil Na supply. In the incubation experiment, soil exchangeable and Colwell K showed a non-significant increase with addition of Na. However, for a pot with 6 kg of soil, the change in Colwell K was equivalent to around 18 mg in the 50 mg Na/kg treatment and could have provided 6 mg of extra K^+^ to each plant in the 3-plant pots, which would account for part of the increased shoot K content in the Na-added plants. From the present results, it would be premature to conclude that Na stimulation of wheat growth in K-deficient plants is unrelated to the increased K availability in soil. Interestingly, previous studies on Na stimulation of plant growth in K-deficient plants did not consider increased K uptake from soil as an explanation for the response: they focussed on Na substitution of K functions. In a solution culture experiment by Box and Schachtman (2000), where Na effects on K availability in the root zone would be absent, there was no evidence of enhanced K^+^ content in wheat due to Na supply, even though there was an increase in wheat growth due to external Na^+^, i.e., according to them the positive effect of Na at low K can be largely attributed to substitution of Na^+^ in wheat K functions and direct effects of Na^+^ on growth. As explained above, Na substitution of K in shoots of wheat in the present experiment was unlikely because the shoot Na concentrations were too low to provide any significant replacement of the osmotic effects of K in vacuoles or in other functions of K^+^. By contrast, the increase in root Na^+^ was more than sufficient to replace osmotic functions of K^+^ in roots.

The increase in root Na concentration may stimulate root elongation of K-deficient plants (Ali et al., 2009) by turgor effects on cell expansion. Whether an increase in root elongation could contribute to increase root K uptake is unclear and there is no direct evidence in the present study to address this question. Such an effect is more likely to be expressed in soil where root elongation has a major role in determining nutrient uptake by providing access to additional nutrient supply (Barber and Silberbush, 1984). It is unlikely the Na substitution of K in roots would directly increase root growth because their dry matter increase would be limited by inadequate assimilate supply to roots under low K supply. Hence, it is proposed that the stimulation of root growth by low to moderate Na is mediated in shoots, probably by increased photosynthesis leading to greater assimilate supply to roots. The increase in root growth in turn could allow for increased K uptake by roots.

Potassium efficient cultivars were more salt tolerant than K-inefficient cultivars in the order: Cranbrook>Wyalkatchem>Gutha>Gamenya, in terms of shoot dry weight (Genc et al., 2007). However, K-efficient cultivars mostly had similar K^+^/Na^+^ ratio to K-inefficient cultivars. This is consistent with an earlier study where K^+^/Na^+^ ratio did not explain the variation in salt tolerance among wheat cultivars (Genc et al., 2007). In contrast, the ability of plants to maintain a high K^+^/Na^+^ ratio was positively correlated with salt tolerance in other studies (Wu et al., 1996; Chen et al., 2007; Shabala and Cuin, 2008; Cuin et al., 2009). Cuin et al. (2008) emphasized that Na^+^ exclusion is not a sufficient tool for salt tolerance but the ability of roots to retain K^+^ correlated better with salt tolerance in wheat. Moreover, a recent study in wheat suggests that salt tolerant cultivars have an enhanced ability to sequester Na^+^ into vacuoles of root cells, whereas in sensitive cultivars large quantities of Na^+^ are located in the root cell cytosol (Cuin et al., 2011). In this study, *cv*. Cranbrook was least effective in retaining root K under increasing Na supply among the cultivars. Cultivars may differ in the extent of Na translocation to shoots. The substitution of K^+^ by Na^+^ in cereals is likely to be influenced not only by plant K status, but also by the potential of cultivar to accumulate significant Na concentrations in their shoots, as emphasized for the salt tolerant barley *cv.* CM72 (Ma et al., 2011), or in roots as with wheat in the present study.

In the present study, the K-use efficiency of wheat cultivars studied across a range of Na levels from no added Na up to toxic levels was consistent with the ranking of cultivars for K-use efficiency by Damon and Rengel (2007). However, there has been little information reported on the role of Na supply in K-use efficiency in wheat. According to this study, K-efficient cultivars Wyalkatchem and Cranbrook had higher response to low to moderate Na supply relative to K-inefficient cultivars Gutha and Gamenya. In contrast to the suggestion by Rengel and Damon (2008) that increased capacity to substitute Na^+^ for K^+^ may be a mechanism underlying K-use efficiency in wheat, we found that Na stimulated greater K uptake in K-efficient cultivars. The main mechanism identified by Damon and Rengel (2007) for K efficiency in wheat cultivars like Wyalkatchem was greater utilization efficiency of shoot K rather than greater K uptake. In the present study, there was greater K uptake by K-efficient cultivars or greater K content in shoots with low to moderate Na supply. The stimulation of photosynthesis, stomatal conductance and transpiration efficiency and root dry weight were greater in the K-efficient cultivars. This is consistent with greater utilization efficiency of shoot K in the K-efficient cultivars leading to greater photosynthesis and hence roots dry weight response to shoot K. Given this explanation the weak responses in shoot dry weight to low to moderate Na are surprising. There was alleviation of K deficiency symptoms in old leaves by low to moderate Na. However, since the symptoms only appeared at 6 weeks after sowing and the shoots were harvested at 8 weeks, it is possible that the shoot response lagged behind that of roots and given more time would have been more substantial.

The stimulation of root growth to a greater extent than shoot growth in wheat by low to moderate Na in low K plants may have greater significance when the crop is under stress in the field than in the present well-watered pot experiment. There should be a direct benefit from an increased root mass under drought stress particularly in K-deficient wheat for which depressed root growth is a characteristic symptom (Ma et al., 2011, 2013).

In summary, wheat cultivars differing in K-use efficiency varied in response to soil K and Na supply. When supplied with low to moderate Na under K deficiency, positive responses in K uptake, leaf photosynthesis, stomatal conductance, and root dry weight were observed in all four cultivars, particularly in K-efficient cultivars. In contrast to previous findings, we conclude that low to moderate Na stimulated increase in shoot K uptake by wheat, which particularly in K-efficient cultivars promoted photosynthesis and root growth and further access to soil K. In the present study, the shoot Na concentrations at low to moderate Na supply to soil were too low to feasibly substitute for biophysical functions of K in the shoot. Four mechanisms are proposed to explain the increased K uptake in shoots of wheat by low to moderate Na supply, but further studies are needed to clarify the relative contribution of each mechanism to the growth stimulation.

## Conflict of Interest Statement

The authors declare that the research was conducted in the absence of any commercial or financial relationships that could be construed as a potential conflict of interest.

## References

[B1] Ali L. Rahmatullah Ranjha A. M. Aziz T. Maqsood M. A. Ashraf M. (2006). Differential potassium requirement and its substitution by sodium in cotton genotypes. *Pak. J. Agric. Sci.* 43 108–113.

[B2] Ali L. Rahmatullah M. Maqsood A. Kanwal S. Ashraf M. Hannan A. (2009). Potassium substitution by sodium in root medium influencing growth behaviour and potassium efficiency in cotton genotypes. *J. Plant Nutr.* 32 1657–1673 10.1080/01904160903150917

[B3] Anschütz U. Becker D. Shabala S. (2014). Going beyond nutrition: regulation of potassium homoeostasis as a common denominator of plant adaptive responses to environment. *J. Plant Physiol.* 171 670–687 10.1016/j.jplph.2014.01.009 24635902

[B4] Barber S. A. Silberbush M. (1984). “Plant root morphology and nutrient uptake,” in *Roots, Nutrients and Water Influx, and Plant Growth* eds Barber S. A. Bouldin D. R. (Madison, WI: American Society of Agronomy), 65–87.

[B5] Benito B. Haro R. Amtmann A. Cuin T. A. Dreyer I. (2014). The twins K^+^ and Na^+^ in plants. *J. Plant Physiol.* 171 723–731 10.1016/j.jplph.2013.10.014 24810769

[B6] Box S. Schachtman D. P. (2000). The effect of low concentrations of sodium and potassium uptake and growth of wheat. *Aust. J. Plant Physiol.* 27 175–182.

[B7] Chen Z. Newman I. Zhou M. Mendham N. Zhang G. Shabala S. (2005). Screening plants for salt tolerance by measuring K^+^ flux: a case study for barley. *Plant Cell Environ.* 28 1230–1246 10.1111/j.1365-3040.2005.01364.x

[B8] Chen Z. H. Zhou M. X. Mendham N. J. Newman I. A. Zhang G. P. Shabala S. (2007). Potassium and sodium relations in salinized barley tissues as a basis of differential salt tolerance. *Funct. Plant Biol.* 34 150–162 10.1071/FP06237 32689341

[B9] Colwell J. D. (1963). The estimation of phosphorus fertiliser requirements of wheat in Southern New South Wales by soil analysis. *Aust. J. Exp. Agric. Anim. Husb.* 3 190–198 10.1071/EA9630190

[B10] Cuin T. A. Betts S. A. Chalmandrier R. Shabala S. (2008). A root’s ability to retain K^+^ correlates with salt tolerance in wheat. *J. Exp. Bot.* 59 2697–2706 10.1093/jxb/ern128 18495637 PMC2486465

[B11] Cuin T. A. Bose J. Stefano G. Jha D. Tester M. Mancuso S. (2011). Assessing the role of root plasma membrane and tonoplast Na^+^/H^+^ exchangers in salinity tolerance in wheat: in planta quantification methods. *Plant Cell Environ.* 34 947–961 10.1111/j.1365-3040.2011.02296.x 21342209

[B12] Cuin T. A. Tian Y. Betts S. A. Chalmandrier R. Shabala S. (2009). Ionic relations and osmotic adjustment in durum and bread wheat under saline conditions. *Funct. Plant Biol.* 36 1110–1119 10.1071/FP09051 32688722

[B13] Damon P. M. Rengel Z. (2007). Wheat genotypes differ in potassium efficiency under glasshouse and field conditions. *Aust. J. Agric. Res.* 58 816–825 10.1071/AR06402

[B14] Degl’Innocenti E. Hafsi C. Guidi L. Navari-Izzo F. (2009). The effect of salinity on photosynthetic activity in potassium-deficient barley species. *J. Plant Physiol.* 166 1968–1981 10.1016/j.jplph.2009.06.013 19604600

[B15] Flowers T. J. Dalmond D. (1992). Protein synthesis in halophytes: the influence of potassium, sodium and magnesium in vitro. *Plant Soil* 146 153–161 10.1007/bf00012008

[B16] Gattward J. N. Almeida A. A. Souza J. O. Gomes F. P. Kronzucker H. J. (2012). Sodium-potassium synergism in *Theobroma cacao*: stimulation of photosynthesis, water-use efficiency and mineral nutrition. *Physiol. Plant.* 146 350–362 10.1111/j.1399-3054.2012.01621.x 22443491

[B17] Genc Y. McDonald G. K. Tester M. (2007). Reassessment of tissue Na^+^ concentration as a criterion for salinity tolerance in bread wheat. *Plant Cell Environ.* 30 1486–1498 10.1111/j.1365-3040.2007.01726.x 17897418

[B18] George M. S. Lu G. Zhou W. (2002). Genotypic variation of K uptake and utilization efficiency in sweet potato. *Field Crops Res.* 77 7–15 10.1016/S0378-4290(02)00043-6

[B19] Huang L. Bell R. W. Dell B. Woodward J. (2004). Rapid nitric acid digestion of plant material with an open-vessel microwave system. *Commun. Soil Sci. Plant Anal.* 35 427–440 10.1081/CSS-120029723

[B20] Idowu M. K. Aduayi E. A. (2007). Sodium-potassium interaction on growth, yield and quality of tomato in ultisol. *J. Plant Interact.* 2 263–271 10.1080/17429140701713803

[B21] Ma Q. Bell R. Brennan R. (2011). Moderate sodium has positive effects on shoots but not roots of salt-tolerant barley grown in a potassium-deficient sandy soil. *Crop Pasture Sci.* 62 972–981 10.1071/CP11162

[B22] Ma Q. Scanlan C. Bell R. Brennan R. (2013). The dynamics of potassium uptake and use, leaf gas exchange and root growth throughout plant phenological development and its effects on seed yield in wheat (*Triticum aestivum*) on a low-K sandy soil. *Plant Soil* 373 373–384 10.1007/s11104-013-1812-z

[B23] Marschner H. (1995). *Mineral Nutrition of Higher plants.* London: Academic Press.

[B24] Marschner H. Kuiper P. J. C. Kylin A. (1981). Genotypic differences in the response of sugar beet plants to replacement of potassium by sodium. *Physiol. Plant.* 51 239–244 10.1111/j.1399-3054.1981.tb02705.x

[B25] Mäser P. Gierth M. Schroeder J. I. (2002). Molecular mechanisms of potassium and sodium uptake in plants. *Plant Soil* 247 43–54 10.1023/a:1021159130729

[B26] Moore G. (2004). “Soil guide- A Handbook for understanding and managing agricultural soils,” in *Potassium* Vol. 4343 ed. Edwards N. (Perth: Department of Agriculture, Western Australia), 176–180.

[B27] Mundy G. N. (1983). Effects of potassium and sodium concentrations on growth and cation accumulation in pasture species grown in sand culture. *Aust. J. Agric. Res.* 34 469–481 10.1071/AR9830469

[B28] Pi Z. Stevanato P. Yv L. H. Geng G. Guo X. L. Yang Y. (2014). Effects of potassium deficiency and replacement of potassium by sodium on sugar beet plants. *Russ. J. Plant Physiol.* 61 224–230 10.1134/s1021443714020101

[B29] Rengel Z. Damon P. M. (2008). Crops and genotypes differ in efficiency of potassium uptake and use. *Physiol. Plant.* 133 624–636 10.1111/j.1399-3054.2008.01079.x 18397208

[B30] Reuter D. J. Edwards D. G. Wilhelm N. S. (1997). “Temperate and tropical crops,” in *Plant Analysis- an Interpretation Manual* eds Reuter D. J. Robinson J. B. (Collingwood, VIC: CSIRO Australia).

[B31] Römheld V. Kirkby E. (2010). Research on potassium in agriculture: needs and prospects. *Plant Soil* 335 155–180 10.1007/s11104-010-0520-1

[B32] Rubio F. Gassmann W. Schroeder J. I. (1995). Sodium-driven potassium uptake by the plant potassium transporter HKT1 and mutations conferring salt tolerance. *Science* 270 1660–1663 10.1126/science.270.5242.1660 7502075

[B33] Searle P. L. (1984). The Berthelot or indophenol reaction and its use in the analytical chemistry of nitrogen- a review. *Analyst* 109 549–568 10.1039/an9840900549

[B34] Shabala S. Cuin T. A. (2008). Potassium transport and plant salt tolerance. *Physiol. Plant.* 133 651–669 10.1111/j.1399-3054.2007.01008.x 18724408

[B35] Subbarao G. V. Ito O. Berry W. L. Wheeler R. M. (2003). Sodium- a functional plant nutrient. *Crit. Rev. Plant Sci.* 22 391–416.

[B36] Tahal R. Mills D. Heimer Y. Tal M. (2000). The relation between low K^+^/Na^+^ ratio and salt-tolerance in the wild tomato species *Lycopersicon pennellii*. *J. Plant Physiol.* 157 59–64 10.1016/S0176-1617(00)80136-4

[B37] Terry N. Ulrich A. (1973). Effects of potassium deficiency on the photosynthesis and respiration of leaves of sugar beet. *Plant Physiol.* 51 43–47 10.1104/pp.51.1.43 16658409 PMC366345

[B38] Walker D. J. Cerdã A. Martãnez V. (2000). The effects of sodium chloride on ion transport in potassium-deficient tomato. *J. Plant Physiol.* 157 195–200 10.1016/S0176-1617(00)80190-X

[B39] Walkley A. Black I. A. (1934). An examination of the Degtjareff method for determining soil organic matter and a proposed modification of the chromic acid titration method. *Soil Sci.* 37 29–38 10.1097/00010694-193401000-00003

[B40] Wu S. J. Ding L. Zhu J. K. (1996). SOS1, a genetic locus essential for salt tolerance and potassium acquistion. *Plant Cell* 8 612–627 10.1105/tpc.8.4.617 PMC161124 12239394

[B41] Zhao D. Oosterhuis D. M. Bednarz C. W. (2001). Influence of potassium deficiency on photosynthesis, chlorophyll content, and chloroplast ultrastructure of cotton plants. *Photosynthetica* 39 103–109 10.1023/a:1012404204910

